# Long non-coding RNA MEG3 mediates the miR-149-3p/FOXP3 axis by reducing p53 ubiquitination to exert a suppressive effect on regulatory T cell differentiation and immune escape in esophageal cancer

**DOI:** 10.1186/s12967-021-02907-1

**Published:** 2021-06-17

**Authors:** Qi-Rong Xu, Jian Tang, Hong-Ying Liao, Ben-Tong Yu, Xiang-Yuan He, Yu-Zhen Zheng, Sheng Liu

**Affiliations:** 1 Department of Thoracic Surgery, The First Affiliated Hospital of Nanchang University, Nanchang, 330006 People’s Republic of China; 2 Department of Thoracic Surgery, The Sixth Affiliated Hospital, Sun Yat-sen UniversityMedical University, No. 26, Erheng Road, Yuancun, Tianhe District, Guangzhou, 510655 Guangdong Province P. R. China

**Keywords:** Esophageal cancer, LncRNA MEG3, MDM2, p53, miR-149-3p, FOXP3, Ubiquitination, Regulatory T cell differentiation, Immune escape

## Abstract

**Background:**

Long non-coding RNA (lncRNA) maternally expressed gene 3 (MEG3) has been implicated in the progression of esophageal cancer (EC). However, the specific mechanism of the involvement of MEG3 in EC development in relation to the regulation of immune escape remains uncertain. Thus, the aim of the current study was to investigate the effect of MEG3 on EC via microRNA-149-3p (miR-149-3p).

**Methods:**

Gain- and loss-of-function experiments were initially performed in EC cells in addition to the establishment of a 4-nitroquinoline 1-oxide-induced EC mouse model aimed at evaluating the respective roles of forkhead box P3 (FOXP3), MEG3, miR-149-3p, mouse double minute 2 homolog (MDM2) and p53 in T cell differentiation and immune escape observed in EC.

**Results:**

EC tissues were found to exhibit upregulated FOXP3 and MDM2 while MEG3, p53 and miR-149-3p were all downregulated. FOXP3 was confirmed to be a target gene of miR-149-3p with our data suggesting it reduced p53 ubiquitination and degradation by means of inhibiting MDM2. P53 was enriched in the promoter of miR-149-3p to upregulate miR-149-3p. The overexpression of MEG3, p53 or miR-149-3p or silencing FOXP3 was associated with a decline in CD25^+^FOXP3^+^CD4^+^ T cells, IL-10^+^CD4^+^ T cells and IL-4^+^CD4^+^ T cells in spleen tissues, IL-4, and IL-10 levels as well as C-myc, N-myc and Ki-67 expression in EC mice.

**Conclusion:**

Collectively, MEG3 decreased FOXP3 expression and resulted in repressed regulatory T cell differentiation and immune escape in EC mice by upregulating miR-149-3p via MDM2-mediated p53.

**Supplementary Information:**

The online version contains supplementary material available at 10.1186/s12967-021-02907-1.

## Background

As one of the least investigated but significantly lethal malignancies on a global scale, esophageal cancer (EC) has been well documented to be a particularly aggressive cancer often associated with a poor survival rate, ranking 6th in terms of cancer-associated fatalities [1]. Although squamous cell carcinoma (SCC) remains the foremost EC subtype, the incidence of esophageal adenocarcinoma has been reported to have increased over the past 4 decades [2]. The two-main histological subtypes of EC have distinct differences in terms of their respective risk factors which has an effect on their incidence and distribution [3]. The immune system serves as a crucial regulator of tumor biology, which possesses the capacity to both support or repress tumor development, growth, invasion and metastasis [4]. Tumor escape from immune-check system represents a distinctly different challenge from that of conventional drug resistance [5]. Evidence has been presented that inefficient tumor rejection is not only the passive consequence of insufficient effector cells, but also a form of active counterattack exerted by the tumor by inducing immunosuppression mechanism to protect them from being eradicated. Impairment of the immune response to cancer arises primarily due to an imbalance between immune activation and immunosuppression [6]. Moreover, immune escape remains a crucial factor for tumor survival, but in turn presents promising possibilities for tumor treatment, highlighting the need for further investigation into the mechanisms underpinning tumor immune escape [7]. Therefore, it is imperative to identify molecular targets associated with immune escape that may be exploited as therapeutic targets for EC.

Accumulating evidence continues to emphasize the effects of long non-coding RNAs (lncRNAs), such as SNHG1, NKILA and UCA1, on tumor immune escape in cancers [8–10]. As an imprinted gene with maternal expression, maternally expressed gene 3 (MEG3) has been reported to exert various anti-oncogenic effects by repressing angiogenesis [11]. Furthermore, MEG3 downregulation has been linked to poor prognosis in patients with esophageal SCC [12]. Also, dysregulated expression of MEG3 leads to the downregulation of ubiquitination ligase mouse double minute 2 homolog (MDM2) in HCT116 and U2OS cells, triggering p53 degradation [13]. MEG3 increases p53 protein level by downregulating MDM2. Notably, p53 has been reported to be enriched in microRNA-149-3p (miR-149-3p) promoter to activate miR-149-3p [14]. A previous study suggested that miR-149 could promote the sensitivity of EC cells to cisplatin [15]. The Starbase database was explored to predict the targeting relationship between miR-149-3p and forkhead box P3 (FOXP3). The overexpression of FOXP3 has been linked with poor EC prognosis and has been suggested to promote immune escape in cholangiocarcinoma [16, 17]. The aforementioned findings contributed to our hypothesis regarding the complex regulation of MEG3 in EC via p53/miR-149-3p/FOXP3 axis. Hence, through the use of EC clinical samples and cells as well as an EC mouse model, we set out to investigate this hypothesis with the objective of identifying potential novel therapeutic targets for EC.

## Methods

### Ethics approval and consent to participate

The study was conducted with the approval of the ethics committee of The First Affiliated Hospital of Nanchang University (Nanchang, China). The study was performed in strict accordance with the *Declaration of Helsinki*. All patients were signed informed consent documentation prior to enrolment, in addition to ethical agreements obtained from all donors or their relatives by written informed consent. All animal experiments were conducted in adherence with the principles listed by the National Institutes of Health Guide for the Care and Use of Laboratory. Extensive efforts were made to minimize pain inflicted on the animals in the current study.

### Clinical sample collection

EC and paracancerous tissues were obtained from 56 EC confirmed patients at tumor-node-metastasis (TNM) III stage at The First Affiliated Hospital of Nanchang University from 2016 to 2018. All specimens were examined by means of endoscopy and biopsy.

Peripheral blood samples were collected from 28 EC patients undergoing cardiothoracic surgery at the Oncology Department of The First Affiliated Hospital of Nanchang University between June 2016 to June 2018 prior to treatment with radiotherapy, systematic chemotherapy or surgery. All patient diagnoses were confirmed by pathology, cytology, and imaging examination. Peripheral blood obtained from 25 healthy blood donors was regarded as the controls.

### EC mice model establishment and grouping

A total of 170 male C57BL/6J mice (animal experimentation license SCXK (Hunan) 2019–0004) aged 6 weeks were recruited to establish an EC model using 4-Nitroquinoline 1-oxide (4-NQO, Shanghai, China). Briefly, 1 mg/mL stock solution was solubilized in propylene glycol, diluted by the drinking water to the final concentration of 100 μg/mL, and immediately fed to mice. The entire process was conducted under dartk conditions in order to prevent degradation. All mice were permitted to drink carcinogens for 16 weeks and subsequently provided with free access to water for the next 6 weeks. All mice were evaluated on a regular basis with records of their weight, toxicity, disease and any abnormalities maintained. All mice were euthanized on the 22nd week for further pathological and molecular assessment [18, 19]. The survival and mental state of the mice was examined on a daily basis. From the 1st week to the 15th week, 1.5 × 10^9^ TU lentivirus was injected into the tail vein of the mice three times per week. The plasmid construction, sequencing identification, virus packaging and titer detection of lentivirus were all performed by Shanghai Genechem Co., Ltd. (Shanghai, China). After the experiment, the survival rate and cancer induction success rate of mice were calculated based on the following formula: survival rate = the number of surviving mice in each group/the total number of mice in each group × 100%, and the cancer induction rate = the number of successfully cancer-induced mice in each group/the total number of in each group × 100%. The survival rate and the cancer induction success rate was confirmed to be 100%. Ten normal mice were adopted as the controls, while the EC-induced mice were injected with lentivirus of short hairpin (sh)-negative control (NC), sh-FOXP3, NC-agomir, miR-149-3p-agomir, miR-149-3p-agomir + overexpression (oe)-NC, miR-149-3p-agomir + oe-FOXP3, oe-NC, oe-p53, oe-p53 + NC-inhibitor, oe-p53 + miR-149-3p-inhibitor, oe-MEG3, oe-MEG3 + sh-NC, oe-MEG3 + sh-p53, oe-MEG3 + oe-NC or oe-MEG3 + oe-FOXP3 (n = 10).

### Preparation of spleen single cell suspension

After the mice had been euthanized, their spleens were removed and placed in a culture dish. After the surface was repeatedly punctured using the syringe needle, the cell suspension was collected and filtered through a 200-mesh sieve to prepare single cell suspension. The suspension was centrifuged at 400×*g* for 5 min. The cell precipitate was suspended in RPMI-1640 medium. The number of living cells was then counted, after which the cell concentration was adjusted to 5 × 10^6^ cells/mL for subsequent experiments.

### Flow cytometry

The cells were re-suspended in staining buffer (BD biosciences, Franklin Lakes, NJ, USA) and stained with antibodies (Abcam Inc., Cambridge, UK) against CD4 (ab237722, 1 μg/mL), CD25 (ab210332, 5 μg/mL), FOXP3 (ab215206, 0.5 μg/mL), interleukin-10 (IL-10, ab189392, 10 μg/mL) and IL-4 (ab11524, 10 μg/mL). Peripheral blood mononuclear cells (PBMCs) from human blood were purified via density gradient centrifugation, and stimulated with 10 ng/mL PMA and 1 μM Ionomycin over a period of 6 h. The PBMCs were subsequently stained with antibodies against CD4 (ab133616, 1 μg/mL, Abcam Inc.), CD25 (#43212, 10 μg/mL, CST, Framingham, MA, USA) and FOXP3 (ab215206, 0.5 μg/mL, Abcam Inc.) for 30 min. Cells with different signals were detected using a flow cytometer BD FACSCantoII (BD biosciences) and analyzed using Flow Jo software.

### EC cell transfection

AKR cells (Shanghai Institute of cell research, Chinese Academy of Sciences, Shanghai, China), were cultured in Dulbecco’s Modified Eagle Medium (DMEM, Gibco life technologies, Grand Island, NY, USA) containing 10% fetal bovine serum (FBS) and 1% penicillin–streptomycin (Gibco life technologies) at 37 °C with 5% CO_2_. The AKR cells were trypsinized and passaged at ratio of 1:3 on three separate occasions. The AKR cells were seeded into a 150-mm dish. After cell confluence had reached 70–80%, the medium was renewed with serum-free medium. AKR cells were transfected with plasmids containing mimic-NC, miR-149-3p mimic, inhibitor-NC, miR-149-3p inhibitor, sh-NC, sh-MDM2, oe-NC, oe-MEG3, oe-NC + sh-NC, oe-MEG3 + sh-NC and oe-MEG3 + sh-p53 using Lipofectamine 2000 (Invitrogen Inc., Carlsbad, CA, USA). All the aforementioned plasmids were constructed by Shanghai Genechem Co., Ltd. The morphological observations of the AKR and HEK293T cells are depicted in Additional file: 1 Figure S1.

### Dual luciferase reporter assay

FOXP3 3'untranslated region (UTR) reporter gene plasmid and mutant plasmids with mutation of miR-149-3p binding site were constructed, namely PmirGLO-FOXP3-wild type (WT) and PmirGLO-FOXP3-mutant (MUT). Reporter plasmids were co-transfected in an independent manner with miR-149-3p-mimic and NC-mimic into HEK293T cells at the 3rd passage for 24 h. The cells were lysed and centrifuged at 12000 rpm for 1 min, after which the supernatant was collected. The luciferase activity was detected using a Dual-Luciferase® Reporter Assay System (E1910, Promega Corporation, Madison, WI, USA), which was equal to the ratio between firefly luciferase and Renilla luciferase.

### Chromatin immunoprecipitation (ChIP)

When the AKR cell density was confirmed to have reached 1 × 10^6^ cells in a 10 cm culture dish, the cells were fixed in 1% formaldehyde for 10 min at 37 °C after the medium had been removed. The fixation was terminated by 5-min culture on ice. Cell precipitate was obtained after digestion and centrifugation. The cells were then re-suspended in 200 μL sodium dodecyl sulfate (SDS) lysis buffer and placed on ice for 10 min to facilitate cross-linking. Chromatin DNA was sheared on ice via ultra-sonication. The supernatant was harvested following 10-min cell centrifugation at 14000 rpm and 4 °C. The cells were diluted with ChIP diluting buffer containing protease inhibitor, and cultured using a blocking solution at 4 °C for 30 min. After the cells had been centrifuged at 1000 rpm and 4 °C, a small amount of the supernatant was employed as the input while the remainder was supplemented with NC anti-rabbit immunoglobulin G (IgG, Abcam Inc.) antibody, with anti-rabbit p53 antibody (Abcam Inc.) added to the leftover supernatant. The entire supernatant was then cultured at 4 °C overnight and added with cross-linked agar, followed by 1-h culture at 4 °C and collection of the complex. The supernatant was discarded after 1 min centrifugation at 1000 rpm at 4 °C, after which the precipitant was eluted using an elution buffer. Next, 20 μL of 5 mol/L NaCl was added to the eluted supernatant and input DNA and subsequently placed into a water bath at 65 °C for 4 h for de-crosslinking. The samples were digested with protease K to remove protein followed by purification and DNA collection. The expression of the combined miR-149-3p promoter DNA was determined by reverse transcription quantitative polymerase chain reaction (RT-qPCR) with the recovered DNA employed as the template.

### Co-immunoprecipitation (Co-IP)

After a 48 h period of transfection, the cells were placed on ice. IP lysis solution was added to the cells in each well after the medium had been removed. The cells were scraped using a cell scraper, and spread evenly using a pipette followed by transfer to an eppendorf (EP) tube. The cells were subsequently lysed on ice for 30 min, followed by centrifugation at 800 rpm and 4 °C for 20 min. The supernatant was then transferred into a new EP tube, with the protein concentration determined using a bicinchoninic acid (BCA) method. The 1 mg protein in each sample was brought to 500 μL with IP lysis solution. Next, 500 μL of each sample was added with primary antibodies and incubated overnight at 4 °C on a mute mixer, respectively. Each tube was supplemented with 20 μL protein A + G beads in the morning of the next day, followed by culture for 2 h. After the impurities had been eluted with IP lysis, the samples were centrifuged at 2500 rpm and 4 °C for 5 min, and washed 5 times. The supernatant was discarded following centrifugation. Next, 20 μL of 2 × loading buffer was assed to each tube and subsequently denatured in metal bath at 100 °C for 5 min. The samples bound to IP were then subjected to western blot analysis using antibodies to p53 (0.5 μg/mL, ab183544, Abcam Inc.) and anti-Ubiquitin (1 μg/mL, ab7780, Abcam Inc.).

### Protein stability test

After the AKR cells had been lysed with Radio-Immunoprecipitation assay (RIPA) lysis (P0013B, Beyotime Biotechnology Co., Shanghai, China), the cells were cultured with PNGase F (G5166-50UN, Sigma-Aldrich Chemical Company, St Louis, MO, USA), and centrifuged at 12000 r/min. Following protein collection, western blot analysis was performed to detect the effect of PNGase F on p53 protein expression.

Next, to evaluate the p53 degradation changes, the cells were treated with tunicamycin (654380, Sigma-Aldrich Chemical Company). Next, 20 μM actinomycin (HY-12320, MCE) was added to AKR to inhibit protein synthesis, followed by protein extraction at 0 min, 30 min, 60 min, 90 min and 120 min for p53 expression detection via western blot analysis. Finally, a degradation curve of p53 was drawn.

### Hematoxylin and eosin (HE) staining

The EC tissue specimens were fixed with 4% formaldehyde for 30–50 min, followed by dehydration, clearing, immersion in wax, embedding and slicing. The tissue slices were flattened and pasted onto coverslips, dried at 45 °C, and dewaxed. The slices were then washed with gradient alcohol and distilled water for 5 min, stained with hematoxylin for 5 min, differentiated in 1% hydrochloric acid ethanol for 3 s, and stained with 5% eosin for about 3 min, followed by dehydration, clearing and sealing. The tissue slices were subsequently observed under a microscope.

### RT-qPCR

TRIZOL (Invitrogen) was employed for total RNA extraction from the cells and tissues. The primers were designed and synthesized by Invitrogen (Additional file 2: Tables S1, S2). The complementary DNA (cDNA) was then synthesized followed the manuals provided by the TaqMan™ MicroRNA Reverse Transcription Kit (4366596, Thermo Fisher Scientific Inc., Waltham, MA, USA) and High-Capacity cDNA Reverse Transcription Kit (4368813, Thermo Fisher Scientific Inc.). RT-qPCR was operated using SYBR®Premix Ex TaqTM (Tli RNaseH Plus) Kit (RR820A, Takara Bio Inc., Tokyo, Japan) on an ABI7500 quantitative PCR instrument (Thermo Fisher Scientific Inc.). The solution was subsequently mixed and promptly centrifuged at 5000 r/min. Real-time fluorescence quantitative PCR instrument (ABI Company, Oyster Bay, NY, USA) was applied for PCR reaction. The relative expression level of mRNA or miRNA was normalized to glyceraldehyde-3-phosphate dehydrogenase (GAPDH) or U6 level and calculated using the 2^−ΔΔCt^ method.

### Western blot analysis

Total protein was extracted from EC cells and tissues through phenylmethylsulfonyl fluoride and protease inhibitors according to the instructions. After the samples had been lysed at 4 °C for 15 min and centrifuged at 15,000 r/min for 15 min, a bicinchoninic acid kit (23227, Thermo Fisher Scientific Inc.) was used to provide an estimation of the protein concentration within the supernatant. After the protein had been separated by polyacrylamide gel electrophoresis methods, the protein on the gel was electroblotted to a polyvinylidene fluoride membrane and subsequently blocked by 5% bovine serum albumin (BSA) at room temperature for 1 h. The membrane was subsequently probed overnight at 4 °C with the diluted primary rabbit antibodies (Abcam Inc.) against p53 (0.5 μg/mL, ab183544), FOXP3 (10 μg/mL, ab215206) and GAPDH (0.5 μg/mL, ab181602). The membrane was then re-probed with horseradish peroxidase labeled goat anti-rabbit IgG secondary antibody (0.1 μg/mL, ab205718, Abcam Inc.) at room temperature for 1.5 h. Developing solution (NCI4106, Pierce, Rockford, IL, USA) was subsequently added to the membrane for color development purposes. Image J 1.48u software (Bio-Rad, Hercules, CA, USA) was employed for protein quantitative analysis, which was expressed as the ratio of the gray value of each protein to that of the internal reference.

### Immunohistochemistry

The prepared paraffin EC tissue slices were baked at 60 °C for 30 min, dewaxed, and hydrated with toluene I, xylene II and gradient alcohol for 5 min respectively. After antigen retrieval with 1 mM tromethamine-ethylene-diamine tetra-acetic acid (PH 8.0) in a microwave, the slices were cooled to room temperature. Endogenous peroxidase blockade was performed using 3% H_2_O_2_-methanol for 10 min at room temperature. Primary rabbit antibodies (Abcam Inc.) against FOXP3 (10 μg/mL, ab215206, Abcam Inc.), C-myc (5 μg/mL, ab32072, Abcam Inc.), N-myc (5 μg/mL, #51705, CST) and Ki-67 (2 μg/mL, ab15580, Abcam Inc.) were added to slices for incubation overnight at 4 °C. The corresponding biotin-labeled secondary goat anti-rabbit IgG antibody (ab150077, 1 μg/mL, Abcam Inc.) was subsequently added and reacted with the slices at room temperature for 10 min. The slices were incubated with streptomycin avidin peroxidase solution at 37 °C for 10 min, and visualized using diaminobenzidine for 20 s, which was stopped in water. The slices were counterstained with hematoxylin and rinsed with running water to facilitate a return to blue for 15 min. The slices were subsequently immersed in varying concentrations of alcohol for dehydration purposes for a period of 2 min each, cleared in xylene, and mounted in neutral gum. Staining was evaluated under a microscope (CX41, Olympus, Tokyo, Japan). Immunohistochemistry was scored based on a previously reported method [20].

### Enzyme-linked immunosorbent assay (ELISA)

The supernatant of the spleen homogenate was harvested in order to determine the levels of cytokines as per the protocol of the IL-10 (ab108870, Abcam Inc.) and IL-4 (ab100710, Abcam Inc.) ELISA kit. The optical density (OD) value at 450 nm was measured using a totipotent enzyme marker (Synergy 2, Winooski, VT, USA). The standard concentration was utilized as the X-axis and OD value as Y-axis in order to determine the regression equation of the standard curve. The OD value was substituted into the equation to calculate the target protein concentration in the samples.

### Statistical analysis

All measurement data were expressed as the mean ± standard deviation and analyzed by SPSS 21.0 software (IBM, Armonk, NY, USA), with *p* < 0.05 considered to be reflective of statistical significance. In the event the data conformed to normal distribution and homogeneity of variance, data between cancer and paracancerous tissues were compared using a paired *t* test, while other data between two groups were compared using an independent sample *t* test. Comparisons among multiple groups were performed using one way analysis of variance (ANOVA), followed by Tukey's post hoc test, while data at different time points among multiple groups were compared using repeated measures ANOVA, followed by the application of a Bonferroni post hoc test. The correlation between the two factors was analyzed by Pearson correlation coefficient.

## Results

### FOXP3 silencing represses immune escape in EC mice by decreasing regulatory T cell differentiation

**Fig. 1:**
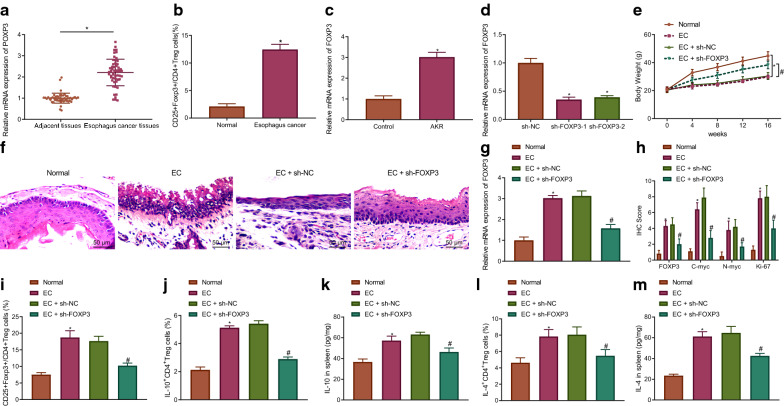
Silencing of FOXP3 inhibits regulatory T cell differentiation to repress immune escape in EC mice. **A** FOXP3 expression in cancer and paracancerous tissues detected by RT-qPCR normalized to GAPDH (n = 56). * *p* < 0.05 vs. paracancerous tissues. **B** The percentage of CD25^+^FOXP3^+^ T cells in CD4^+^ T cells of peripheral blood from EC patients and healthy blood donors assessed by flow cytometry (EC patients: n = 28; healthy blood donors: n = 25). * *p* < 0.05 vs*.* peripheral blood from healthy blood donors. **C** FOXP3 expression in AKR cells detected by RT-qPCR normalized to GAPDH. * *p* < 0.05 vs*.* control cells. **D** The silencing efficiency of sh-FOXP3-1 and sh-FOXP3-2 in AKR cells detected by RT-qPCR normalized to GAPDH. * *p* < 0.05 vs. AKR cells transfected with sh-NC. Normal mice were used as controls, and 4-NQO-induced mice were transfected or not transfected with sh-NC or sh-FOXP3. **E** The weight of mice (n = 10). **F** Pathological changes of tissues in mice measured by HE staining (200×) (n = 10). **G** FOXP3 expression in tissues of mice detected by RT-qPCR normalized to GAPDH (n = 10). **H** FOXP3, C-myc, N-myc and Ki-67 positive protein expression in tissues of mice determined by immunohistochemistry (n = 10). **I** The percentage of CD25^+^FOXP3^+^ T cells in CD4^+^ T cells in spleen of mice assessed by flow cytometry (n = 10). **J** The percentage of IL-10^+^CD4^+^ T cells in spleen of mice assessed by flow cytometry (n = 10). **K** IL-10 level in the spleen of mice assessed by ELISA (n = 10). **L** The percentage of IL-4^+^CD4^+^ T cells in spleen of mice assessed by flow cytometry (n = 10). **M** IL-4 level in the spleen of mice assessed by ELISA (n = 10). * *p* < 0.05 vs*.* normal mice. # *p* < 0.05 vs. EC mice treated with sh-NC. Measurement data were expressed as mean ± standard deviation. Data between cancer and paracancerous tissues were compared by paired *t* test, and data between other two groups were compared by independent sample *t* test. Comparisons among multiple groups were performed using one way analysis of variance (ANOVA), followed by Tukey's post hoc test, and data at different time points among multiple groups were compared by repeated measures ANOVA, followed by Bonferroni post hoc test. The cell experiment was repeated 3 times

In order to ascertain the effect of FOXP3 on the differentiation of regulatory T cells in the tumorigenesis of EC in mice, 4-NQO was utilized to establish EC mouse models. RT-qPCR revealed that the expression of FOXP3 was markedly elevated in the EC tissues (Fig. 1A). The flow cytometry results indicated that the percentage of CD25^+^FOXP3^+^ T cells was increased in the CD4^+^ T cells in peripheral blood of EC patients (Fig. 1B). Meanwhile, RT-qPCR results demonstrated the elevation of FOXP3 expression in the AKR cells. The AKR cells were subsequently transfected with sh-FOXP3-1 and sh-FOXP3-2. sh-FOXP3-1 with better silencing efficiency was selected for follow-up experiments (Fig. 1C, D). The results in Fig. 1E indicated that the weight of the EC mice had decreased more distinctly relative to that of the control mice, which was restored following the silencing FOXP3. Moreover, 4-NQO induced esophageal carcinogenesis in mice, while silencing of FOXP3 inhibited the tumor growth in EC mice (Fig. 1F). The RT-qPCR results revealed that post 4-NQO induction, FOXP3 expression was enhanced in the mice, which was reversed following sh-FOXP3 treatment (Fig. 1G). Immunohistochemistry findings illustrated that 4-NQO triggered an increase in the expression of FOXP3 and malignant markers (C-myc, N-myc and Ki-67) in mice, which was negated after silencing FOXP3 (Fig. 1H). Flow cytometry findings provided evidence demonstrating that the percentage of CD25^+^FOXP3^+^ T cells in CD4^+^ T cells, IL-10^+^CD4^+^ T cells and IL-4^+^CD4^+^ T cells was elevated in the splenic tissues of the mice treated with 4-NQO, which was abrogated by silencing FOXP3 (Fig. 1I–L). ELISA observations indicated that 4-NQO-induced mice had enhanced IL-10 and IL-4 levels in the splenic tissues, which was neutralized following transfection with sh-FOXP3 (Fig. 1K, M). Taken together, the aforementioned results indicate that FOXP3 silencing inhibits regulatory T cell differentiation to suppress immune escape in EC mice.

### miR-149-3p targets FOXP3 and is inversely correlated with FOXP3 expression in EC

**Fig. 2:**
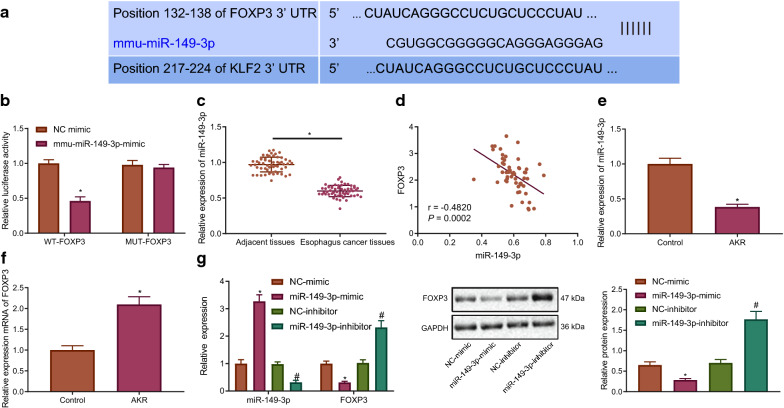
FOXP3 is negatively targeted by miR-149-3p in EC. **A** The binding sites between miR-149-3p and FOXP3 predicted by Starbase. **B** The targeting relationship between miR-149-3p and FOXP3 evaluated by dual luciferase reporter assay. * *p* < 0.05 vs. the treatment of NC-mimic. **C** miR-149-3p expression in cancer and paracancerous tissues detected by RT-qPCR normalized to U6 (n = 56). * *p* < 0.05 vs. paracancerous tissues. **D** The correlation between miR-149-3p and FOXP3 expression in EC tissues. **E** Expression of miR-149-3p in AKR cells detected by RT-qPCR normalized to U6. **F** Protein expression of FOXP3 in AKR cells measured by western blot analysis normalized to GAPDH. * *p* < 0.05 vs. control cells. **G** Protein expression of FOXP3 in different AKR cells measured by western blot analysis normalized to GAPDH. * *p* < 0.05 vs. AKR cells treated with NC-mimic. # *p* < 0.05 vs. AKR cells treated with NC-inhibitor. The measurement data were shown as mean ± standard deviation. Data between cancer and paracancerous tissues were compared by paired *t* test, and data between other two groups were compared by independent sample *t* test. Pearson was used for correlation analysis between miR-149-3p and FOXP3 expression in EC tissues. The cell experiment was repeated 3 times independently

The emphasis of the study was then shifted to elucidate the upstream mechanism of FOXP3 in EC. Initially, Starbase was employed to predict whether FOXP3 was a target of miR-149-3p (Fig. 2A). Moreover, dual luciferase reporter assay revealed that the luciferase activity was markedly diminished in response to WT-FOXP3 while no changes were noted in MUT-FOXP3 (Fig. 2B), highlighting a targeting relationship between miR-149-3p and FOXP3. Furthermore, RT-qPCR results manifested that miR-149-3p expression was lowly expressed in EC tissue than in paracancerous tissue (Fig. 2C). Correlation analysis demonstrated an inverse correlation between miR-149-3p and FOXP3 expression in EC tissues (Fig. 2D). The expression of miR-149-3p exhibited reduced levels in the AKR cells, while the expression of FOXP3 was increased in the AKR cells (Fig. 2E, F). miR-149-3p-mimic reduced but miR-149-3p-inhibitor elevated FOXP3 expression in AKR cells (Fig. 2G). Taken together, the data obtained indicated that FOXP3 was a target gene of miR-149-3p in EC.

### miR-149-3p targets FOXP3 to reduce regulatory T cell differentiation to repress immune escape in EC mice

**Fig. 3:**
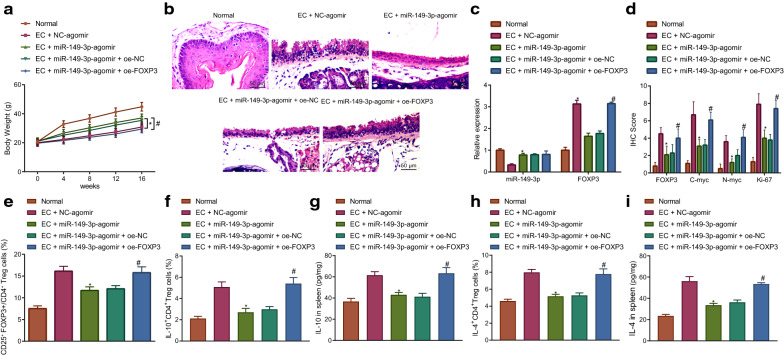
miR-149-3p inhibits regulatory T cell differentiation to repress immune escape in EC mice via FOXP3. Normal mice were used as controls, and EC mice were treated or not treated with NC-agomir, miR-149-3p-agomir, miR-149-3p-agomir + oe-NC or miR-149-3p-agomir + oe-FOXP3. **A** The weight of mice. **B** Pathological changes of tissues in mice measured by HE staining (200×). **C** FOXP3 expression in tissues of mice detected by RT-qPCR normalized to GAPDH. **D** Immunohistochemistry result of detecting FOXP3, C-myc, N-myc and Ki-67 protein expression. **E** The percentage of CD25^+^FOXP3^+^ T cells in CD4^+^ T cells in mice spleen assessed by flow cytometry. **F** The percentage of IL-10^+^CD4^+^ T cells in mice spleen assessed by flow cytometry. **G** IL-10 level in the spleen of mice assessed by ELISA. **H** The percentage of IL-4^+^CD4^+^ T cells in mice spleen assessed by flow cytometry. **I** IL-4 level in the spleen of mice assessed by ELISA. * *p* < 0.05 vs*.* EC mice treated with NC-agomir. # *p* < 0.05 vs. EC mice treated with miR-149-3p-agomir + oe-NC. Measurement data were expressed as mean ± standard deviation. Data between two groups were compared by independent sample *t* test. Comparisons among multiple groups were performed using one way analysis of variance (ANOVA), followed by Tukey's post hoc test, and data at different time points among multiple groups were compared by repeated measures ANOVA, followed by Bonferroni post hoc test. n = 10

The aforementioned results provided evidence of a targeting relationship between miR-149-3p and FOXP3. We subsequently speculated that miR-149-3p targeted FOXP3 to regulate T cell function and inhibit immune escape in EC. 4-NQO was utilized to establish EC mouse models in order to investigate changes to the regulatory T cells. As depicted in Fig. 3A, the weight of EC mice was notably increased following miR-149-3p-agomir treatment, which was normalized in the presence of overexpressed FOXP3. Moreover, miR-149-3p-agomir treatment triggered reduced esophageal carcinogenesis in EC mice, which was neutralized by additional treatment with oe-FOXP3 (Fig. 3B). As demonstrated in the RT-qPCR results, among the EC mice treated with miR-149-3p-agomir exhibited decreased FOXP3 expression, which was reversed by oe-FOXP3 transfection (Fig. 3C). Meanwhile, the immunohistochemistry results indicated that FOXP3, C-myc, N-myc and Ki-67 expression were all reduced in the EC mice treated with miR-149-3p-agomir, which was reversed by additional overexpression of FOXP3 (Fig. 3D). Flow cytometry suggested a reduction in the percentage of CD25^+^FOXP3^+^ T cells in CD4^+^ T cells, IL-10^+^CD4^+^ T cells and IL-4^+^CD4^+^ T cells in spleen tissues of miR-149-3p-agomir-treated EC mice, which was reversed in the presence of overexpressed FOXP3 (Fig. 3E, F, H). Furthermore, ELISA findings indicated that IL-10 and IL-4 levels were diminished in the splenic tissues of miR-149-3p-agomir-treated EC mice, which was rescued in response to additional oe-FOXP3 treatment (Fig. 3G, I). In conclusion, miR-149-3p inhibited regulatory T cell differentiation and immune escape in EC mice by targeting FOXP3.

### p53 represses regulatory T cell differentiation to inhibit immune escape in EC mice by downregulating miR-149-3p-targeted FOXP3

**Fig. 4:**
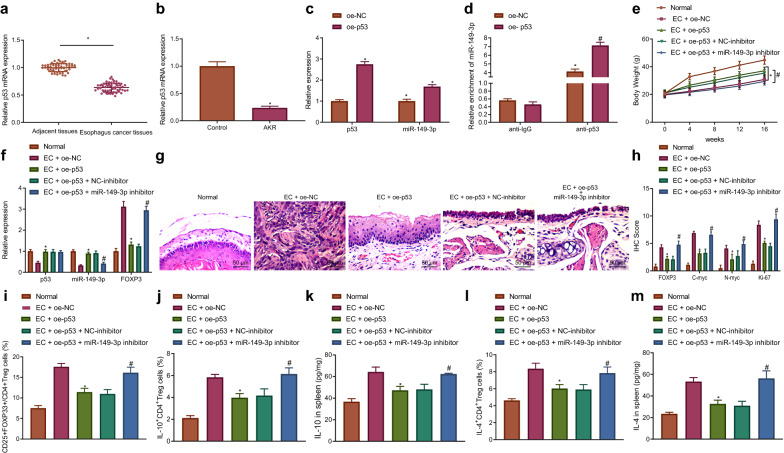
p53-activated miR-149-3p results in suppression of regulatory T cell differentiation and immune escape in EC mice via FOXP3. **A** p53 expression in cancer and paracancerous tissues detected by RT-qPCR normalized to GAPDH (n = 56). * *p* < 0.05 vs. paracancerous tissues. **B** p53 expression in AKR cells detected by RT-qPCR normalized to GAPDH. * *p* < 0.05 vs. control cells. **C** p53 and miR-149-3p expression in AKR cells after overexpression of p53 detected by RT-qPCR normalized to GAPDH and U6. * *p* < 0.05 vs. AKR cells treated with oe-NC. **D** The enrichment of p53 in miR-149-3p promoter measured by ChIP. * *p* < 0.05 vs. IgG. Normal mice were used as controls, and EC mice were treated or not treated with oe-NC, oe-p53, oe-p53 + NC-inhibitor or oe-p53 + miR-149-3p-inhibitor. **E** The weight of mice (n = 10). **F** FOXP3 expression in mice tissues detected by RT-qPCR normalized to GAPDH (n = 10). **G** Pathological changes of mice tissues measured by HE staining (200 ×) (n = 10). **H** Immunohistochemistry result of detecting FOXP3, C-myc, N-myc and Ki-67 protein expression (n = 10). **I** The percentage of CD25^+^FOXP3^+^ T cells in CD4^+^ T cells in mice spleen assessed by flow cytometry (n = 10). **J** The percentage of IL-10^+^CD4^+^ T cells in mice spleen assessed by flow cytometry (n = 10). **K** IL-10 level in mice spleen assessed by ELISA (n = 10). **L** The percentage of IL-4 + CD4 + T cells in mice spleen assessed by flow cytometry (n = 10). **M** IL-4 level in mice spleen assessed by ELISA (n = 10). * *p* < 0.05 vs. EC mice treated with oe-NC. # *p* < 0.05 vs. EC mice treated with oe-p53 + NC-inhibitor. Measurement data were expressed as mean ± standard deviation. Data between cancer and paracancerous tissues were compared by paired *t* test, and data between other two groups were compared by independent sample *t* test. Comparisons among multiple groups were performed using one way analysis of variance (ANOVA), followed by Tukey's post hoc test, and data at different time points among multiple groups were compared by repeated measures ANOVA, followed by Bonferroni post hoc test. The cell experiment was repeated 3 times

We subsequently set out to further ascertain the involvement of p53 in EC immune escape through the miR-149-3p/FOXP3 axis. The RT-qPCR results illustrated that the expression of p53 was significantly diminished in the EC tissues and AKR cells, while the overexpression of p53 in the AKR cells triggered an increase in the expression of miR-149-3p (Fig. 4A–C). ChIP results indicated that p53 was enriched in miR-149-3p promoter (Fig. 4D). The results in Fig. 4E suggested that the weight of p53-overexpressed EC mice was increased, which was restored following the introduction of additional miR-149-3p-inhibitor treatment. As depicted in Fig. 4F, the expression of FOXP3 was diminished in EC mice in response to oe-p53, which was rescued by additional miR-149-3p-inhibitor treatment. Meanwhile, after overexpressing p53, esophageal carcinogenesis was reduced in the EC mice, which was neutralized by additional treatment with miR-149-3p-inhibitor (Fig. 4G). Immunohistochemistry demonstrated that the expression of FOXP3, C-myc, N-myc and Ki-67 was decreased in the EC mice after overexpressing p53, which was reversed by miR-149-3p-inhibitor treatment (Fig. 4H). Flow cytometry results subsequently indicated overexpressed p53 led to a decline in the percentage of CD25^+^FOXP3^+^ T cells in CD4^+^ T cells, IL-10^+^CD4^+^ T cells and IL-4^+^CD4^+^ T cells in splenic tissues of the EC mice, which was normalized following treatment with miR-149-3p-inhibitor (Fig. 4I–L). Additionally, p53-overexpressed EC mice had reduced IL-10 and IL-4 levels in the splenic tissues, which was reversed following additional miR-149-3p-inhibitor treatment (Fig. 4K, M). In summary, p53 decreased the expression of FOXP3 via upregulating miR-149-3p, which ultimately repressed regulatory T cell differentiation and immune escape in EC mice.

### MEG3 regulates miR-149-3p/FOXP3 axis by inhibiting the ubiquitination of p53 via ubiquitination ligase MDM2 in EC cells

**Fig. 5:**
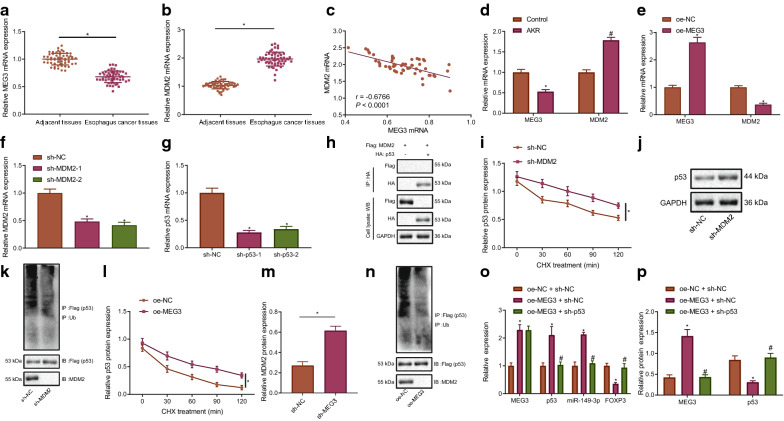
MEG3 suppresses the ubiquitination of p53 by downregulating MDM2 to regulate miR-149-3p/FOXP3 axis in EC cells. **A** MEG3 expression in cancer and paracancerous tissues detected by RT-qPCR normalized to GAPDH (n = 56). **B** MDM2 expression in cancer and paracancerous tissues detected by RT-qPCR normalized to GAPDH (n = 56). * *p* < 0.05 vs. paracancerous tissues. **C** The correlation between MEG3 and MDM2 expression in EC tissues. **D** MEG3 and MDM2 expression in AKR cells detected by RT-qPCR normalized to GAPDH. * *p* < 0.05 *vs.* control cells. **E** mRNA expression of MEG3 and MDM2 in AKR cells after overexpressing MEG3 detected by RT-qPCR normalized to GAPDH. * *p* < 0.05 vs. AKR cells treated with oe-NC. **F** Silencing efficiency of sh-MDM2 in AKR cells determined by RT-qPCR normalized to GAPDH. **G** Silencing efficiency of sh-p53 in AKR cells determined by RT-qPCR normalized to GAPDH. * *p* < 0.05 vs. AKR cells transfected with sh-NC. **H** The binding relationship between MDM2 and p53 determined by Co-IP. * *p* < 0.05 vs. IgG. **I** Effect of MDM2 inhibition on the stability of p53 protein in AKR cells after cycloheximide (CHX, 20 μM) treatment. **J** p53 expression in AKR cells after silencing MDM2 determined by western blot analysis normalized to GAPDH. **K** Western blot analysis result of the ubiquitination of p53 in AKR cells with MDM2 inhibition normalized to GAPDH. * *p* < 0.05 vs. AKR cells transfected with sh-NC. L, Effect of MEG3 overexpression on the stability of p53 protein in AKR cells after cycloheximide (CHX, 20 μM) treatment. **M** p53 expression in overexpressed MEG3 AKR cells determined by western blot analysis normalized to GAPDH. **N** Western blot analysis result of the ubiquitination of p53 in AKR cells with MDM2 overexpression normalized to GAPDH. * *p* < 0.05 *vs.* AKR cells transfected with oe-NC. **O** MEG3, p53, miR-149-3p and FOXP3 expression in different AKR cells measured by RT-qPCR normalized to GAPDH and U6. **P** p53 and FOXP3 expression in different AKR cells measured by western blot analysis normalized to GAPDH. * *p* < 0.05 vs. AKR cells treated with oe-NC + sh-NC. # *p* < 0.05 vs. AKR cells treated with oe-MEG3 + sh-NC. Measurement data were expressed as mean ± standard deviation. Data between cancer and paracancerous tissues were compared by paired *t* test, and data between other two groups were compared by independent sample *t* test. Comparisons among multiple groups were performed using one way analysis of variance (ANOVA), followed by Tukey's post hoc test, and data at different time points among multiple groups were compared by repeated measures ANOVA, followed by Bonferroni post hoc test. The cell experiment was repeated 3 times

We subsequently continued our investigation into whether MEG3 could decreased the ubiquitination of p53 via ubiquitination ligase MDM2. As demonstrated in RT-qPCR results, MEG3 downregulation and MDM2 upregulation were found in the EC tissues (Fig. 5A, B). Meanwhile, a negative correlation between MEG3 and MDM2 was identified in the EC tissues (Fig. 5C). Additionally, the expression of MEG3 was downregulated along with upregulated MDM2 detected in the AKR cells, with overexpressed MEG3 found to decrease the expression of MDM2 in AKR cells (Fig. 5D, E). In addition, sh-MDM2-1 and sh-MDM2-2 deceased MDM2 expression, and that sh-p53-1 and sh-p53-2 reduced p53 expression in AKR cells (Fig. 5F, G). The sh-MDM2-1 and sh-p53-1 with better silencing efficiency were selected for subsequent experiments. Co-IP results indicated that in AKR cells, MDM2 and p53 were interacted with each other (Fig. 5H). Silencing MDM2 (Fig. 5I–K) and overexpressing MEG3 (Fig. 5L–N) decreased the degradation and ubiquitination of p53, but increased p53 protein expression. In the AKR cells, oe-MEG3 treatment elevated p53 and miR-149-3p expression and declined FOXP3 expression, which was reversed by silencing p53 (Fig. 5O, P). The aforementioned results suggested that MEG3 repressed MDM2 to decrease the ubiquitination of p53, thereby regulating miR-149-3p/FOXP3 axis in EC cells.

### MEG3 elevation represses regulatory T cell differentiation and immune escape by inhibiting FOXP3 or elevating p53 in EC mice

**Fig. 6:**
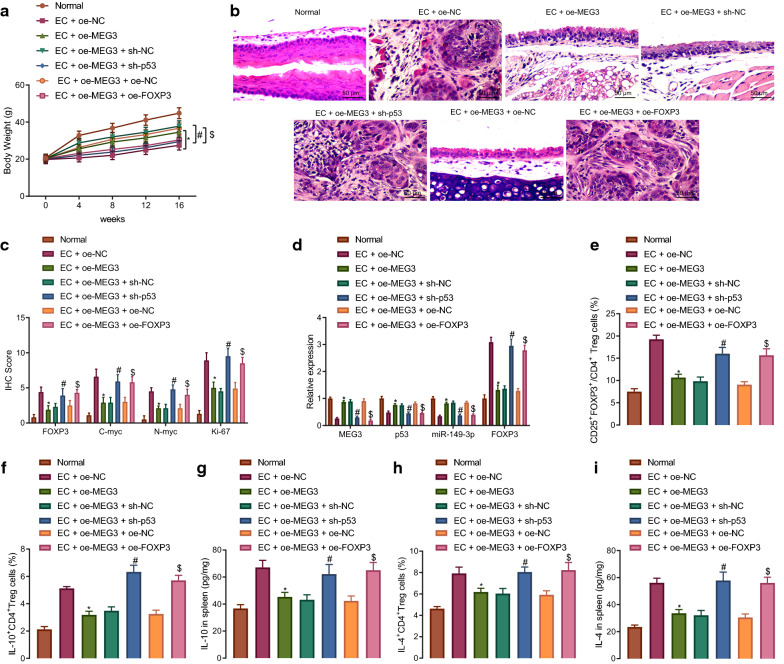
MEG3 suppresses immune escape by decreasing regulatory T cell differentiation through p53/miR-149-3p/FOXP3 axis. Normal mice were used as controls, and EC mice were treated or not treated with oe-NC, oe-MEG3, oe-MEG3 + sh-NC, oe-MEG3 + sh-p53, oe-MEG3 + oe-NC or oe-MEG3 + oe-FOXP3. **A** The weight of mice. **B** Pathological changes of tissues in mice measured by HE staining (200×). **C** Immunohistochemistry result of detecting FOXP3, C-myc, N-myc and Ki-67 protein expression. **D** MEG3, p53, miR-149-3p and FOXP3 expression in mice tissues detected by RT-qPCR normalized to GAPDH and U6. **E** The percentage of CD25^+^FOXP3^+^ T cells in CD4^+^ T cells in mice spleen assessed by flow cytometry. **F** The percentage of IL-10^+^CD4^+^ T cells in mice spleen assessed by flow cytometry. **G** The percentage of IL-4^+^CD4^+^ T cells in mice spleen assessed by flow cytometry. **H** IL-10 level in the spleen of mice assessed by ELISA. **I** IL-4 level in the spleen of mice assessed by ELISA. * *p* < 0.05 vs. EC mice treated with oe-NC. # *p* < 0.05 vs. EC mice treated with oe-MEG3 + sh-NC. $ vs. EC mice treated with oe-MEG3 + oe-NC. Measurement data were expressed as mean ± standard deviation. Data between two groups were compared by independent sample *t* test. Comparisons among multiple groups were performed using one way analysis of variance (ANOVA), followed by Tukey's post hoc test, and data at different time points among multiple groups were compared by repeated measures ANOVA, followed by Bonferroni post hoc test. n = 10

In the following experiment, we further explored whether MEG3 participated in immune escape of EC by inhibiting regulatory T cell differentiation through the p53/miR-149-3p/FOXP3 axis. Firstly, overexpression of MEG3 led to an enhancement in the weight of the EC mice, which was reversed by overexpressing FOXP3 or silencing p53 (Fig. 6A). Moreover, overexpression of MEG3 led to a decrease in esophageal carcinogenesis in EC mice, which was restored following additional treatment with oe-FOXP3 or sh-p53 (Fig. 6B). Meanwhile, immunohistochemistry highlighted a decline in the expression of FOXP3, C-myc, N-myc and Ki-67 in the EC mice following MEG3 overexpression, which was rescued in the presence of overexpressed FOXP3 or silenced p53 (Fig. 6C). RT-qPCR indicated that MEG3 overexpression could bring about an increase in p53 and miR-149-3p expression along with a decrease in FOXP3 expression, which was negated by silencing p53 or overexpressing FOXP3 (Fig. 6D). Flow cytometry exhibited that the percentage of CD25^+^FOXP3^+^ T cells in CD4^+^ T cells, IL-10^+^CD4^+^ T cells and IL-4^+^CD4^+^ T cells was diminished in the splenic tissues of MEG3-overexpressed EC mice, which was repealed following additional treatment with sh-p53 or oe-FOXP3 (Fig. 6E–G). ELISA results indicated that the IL-10 and IL-4 levels were diminished in the splenic tissues of MEG3-overexpressed EC mice, which was neutralized in the presence of sh-p53 or oe-FOXP3 (Fig. 6H, I). Taken together, the aforementioned data suggested that MEG3 could repress regulatory T cell differentiation to decrease immune escape in EC mice via the p53/miR-149-3p/FOXP3 axis.

## Discussion

As one of the most prevalent malignancies and often accompanied by high fatalities, EC poses a significant risk to both the cardiac and pulmonary systems. In spite of commendable treatment developments and treatment delivery methods, EC is still associated with poor survival rates [21]. LncRNAs represent crucial factors in the development of EC [22]. Existing literature has emphasized MEG3 as a tumor-suppressing lncRNA [11]. However, the mechanism of MEG3 in EC is yet to be fully elucidated. Hence, the present study aimed to investigate the specific role of MEG3 in EC via miR-149-3p. Consequently, our data revealed that MEG3 could inhibit immune escape in EC mice by means of downregulating miR-149-3p-targeted FOXP3 via p53.

The initial key observation made during our study revealed that the expression of MEG3 was downregulated in EC. Consistent with our results, MEG3 has been previously reported to be a tumor suppressor in various types of cancers, including that of cervical, gastric and bladder cancers [23–25]. MEG3 downregulation has been noted in esophageal SCC cells, EC cells and tissues [12, 26], all of which was consistent with our data. Our study also uncovered that p53 was downregulated and MDM2 was upregulated in EC tissues, and that MEG3 sustained p53 by inhibiting p53 ubiquitination via MDM2 downregulation in EC cells. Ubiquitination is a general post-transcriptional modification which reduces protein levels of actin cytoskeleton regulatory factors, while certain lncRNAs have been implicated in the process of tumorigenesis and tumor progression through ubiquitination [27]. Additionally, MDM2, an E3 ubiquitin ligase, ubiquitinated p53 to induce its degradation in mouse dental papilla cells [28]. In line with our data, a previous study revealed that MEG3 upregulation suppresses p53 ubiquitination and degradation by downregulating MDM2 to increase the p53 protein level in HCT116 and U2OS cells [13]. Ryuichiro et al*.* concluded that MDM2 is upregulated in esophageal SCC tissues [29]. Meanwhile, previous evidence has been presented indicating that p53 is downregulated in esophageal SCC cells [30]. Additionally, our study uncovered that p53 was enriched in the miR-149-3p promoter to activate miR-149-3p, which was further supported by a study conducted by Jin et al*.* [14]. Furthermore, our study revealed that miR-149-3p was downregulated while FOXP3 was upregulated in EC tissues. Concurrent with our data, previous literature has reported that miR-149 is poorly expressed in EC cells while FOXP3 has exhibited heightened expression in EC cells [15, 16]. FOXP3 was confirmed to be a putative target gene of mIR-149-3p during our investigation. Reports have suggested that miRNAs may contribute to cancer progression by means of mediating gene expression via transcription inhibition and/or promoting degradation of target mRNAs [31]. For instance, miR-149-3p targeted pyruvate dehydrogenase kinases 2 (PDK2) to induce chemosensitivity of colorectal cancer cells [32]. miR-125b promoted autophagy and the efficacy of cisplatin by negatively targeting FOXP3 [33]. These discoveries supported our result that miR-149-3p targeted FOXP3.

Finally, our results demonstrated that MEG3 could suppress immune escape by decreasing regulatory T cell differentiation through p53/miR-149-3p/FOXP3 axis, which was accompanied by diminished levels of IL-4 and IL-10. The proliferation of tumor cells has been shown to influence the immune response to induce myeloid-derived suppressor cells and regulatory T cells, ultimately limiting the efficiency of effective anti-tumor lymphocytes in clearing tumor cells [34]. Regulatory T cells have been implicated in the event of immune escape observed in papillary thyroid cancer [35]. Intestinal inflammation is attenuated by FOXP3^−^ IL-10^−^ regulatory T-cells induced by B-Cells [36]. Meanwhile, the overexpression of FOXP3 has been reported to play a regulatory role in T cell differentiation while inducing immune escape in cholangiocarcinoma cells [17]. Reports have suggested that FOXP3 upregulation heightens the degree of immune escape seen in cervical cancer cells [37].

## Conclusion

**Fig. 7:**
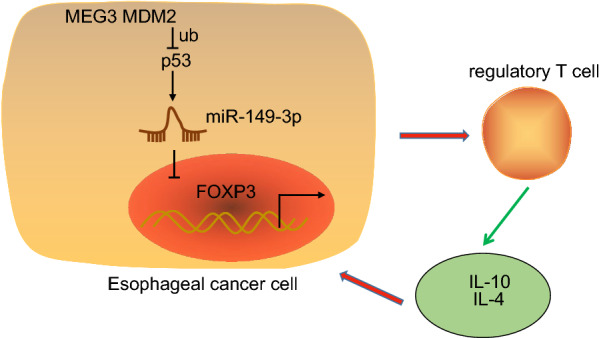
The mechanism of MEG3 in EC. MEG3 was found to downregulate MDM2 to repress p53 ubiquitination and degradation, activating p53 to elevate miR-149-3p expression, which inhibited regulatory T cell differentiation and immune escape in EC mice by targeting FOXP3

Taken together, the key findings of our study provide evidence attesting the anti-oncogenic effect of MEG3 in EC development. Briefly, MEG3 was found to downregulate MDM2 which led to a decrease in p53 ubiquitination and degradation, activating p53 to elevate miR-149-3p expression, which inhibited regulatory T cell differentiation and immune escape in EC mice by targeting FOXP3 (Fig. 7). Our observations provide a platform for understanding the complex mechanisms underpinning the MEG3/MDM2/p53/miR-149-3p/FOXP3 axis in EC progression and provide a potential new therapeutic target for EC. However, further studies need to be performed to determine the potential application for these findings in a clinical setting.

## Supplementary Information

**Additional file 1: Figure S1.** Morphological observation of AKR and HEK293T cells (× 200).

**Additional file 2: Table S1.** Primer sequences of human genes for RT-qPCR. **Table S2.** Primer sequences of mouse genes for RT-qPCR.

## Data Availability

The datasets generated/analyzed during the current study are available.

## Abbreviations

lncRNA: Long non-coding RNA
EC: Esophageal cancer
SCC: Squamous cell carcinoma
MEG3: Maternally expressed gene 3
FOXP3: Forkhead box P3
UTR: Untranslated region
ChIP: Chromatin immunoprecipitation
SDS: Sodium dodecyl sulfate
Co-IP: Co-immunoprecipitation
ELISA: Enzyme-linked immunosorbent assay
