# Supplementary material for: Long non-coding RNA MEG3 mediates the miR-149-3p/FOXP3 axis by reducing p53 ubiquitination to exert a suppressive effect on regulatory T cell differentiation and immune escape in esophageal cancer
Source: J Transl Med. 2021 Jun 17;19:264. doi: 10.1186/s12967-021-02907-1 (PMC8212454; doi:10.1186/s12967-021-02907-1)
Supplement: Supplementary file 2 — Additional file 2: Table S1. Primer sequences of human genes for RT-qPCR. Table S2. Primer sequences of mouse genes for RT-qPCR. [file 12967_2021_2907_MOESM2_ESM.docx]

**Table S1**

Primer sequences of human genes for RT-qPCR

| Genes | Forward (5’-3’) | Reverse (5’-3’) |
| --- | --- | --- |
| p53 | ACCACCATCCACTACAACTACAT | CCAGGACAGGCACAAACAT |
| miR-149-3p | AGGGAGGGACGGGGGCTGTGC | GAGCAGGCTGGAGAA |
| FOXP3 | AGATGGTACAGTCTCTGGAGCAG | AAGTAGTCCATGTTGTGGAGGAA |
| MEG3 | CCACTCCCAGTTCAATTACAGCTC | TAGTGCCCTCGTGAGGTGTAG |
| MDM2 | GACTCCAAGCGCGAAAAC | CAGACATGTTGGTATTGCACATT |
| U6 | CTCGCTTCGGCAGCACA | AACGCTTCACGAATTTGCGT |
| GAPDH | TCAACAGCAACTCCCACTCTTCCA | ACCCTGTTGCTGTAGCCGTATTA |

**Table S2**

Primer sequences of mouse genes for RT-qPCR

| Genes | Forward (5’-3’) | Reverse (5’-3’) |
| --- | --- | --- |
| p53 | GCCTATCCAACGGATCAGAC | ATTTCCCTCAGGTGGATAGA |
| miR-149-3p | GAGGGAGGGACGGGGGCGGTGC | GCGAGCACAGAATTAATACGAC |
| FOXP3 | AGGAGAAAGCGGATACCA | TGTGAGGACTACCGAGCC |
| MEG3 | CTGCCCATCTACACCTCACG | CTCTCCGCCGTCTGCG CTAGGGGCT |
| MDM2 | GGAAGTCGATGGTTGGGAATAG | AGCTGACAGAGAATGATGCTAAA |
| U6 | GCATGACGTCTGCTTTGGA | CCACAATCATTCTGCCATCA |
| GAPDH | AGGTCGGTGTGAACGGATTTG | TGTAGACCATGTAGTTGAGGTCA |
